# Maximum Doses of Medicine

**Published:** 1822-02

**Authors:** 


THE LONDON
### Medical and Physical Journal.

2 OF VOL. XLVII.] FEBRUARY, 1822. [NO. 276.

For many fortunate discoveries in medicine, and for the detection of numerous errors, the world is indebted to the rapid circulation of Monthly Journals; and there never existed any work, to which the Faculty, in Europe and America, were under deeper obligations, than to the Medical and Physical Journal of London, now forming a long, but an invaluable, series.—RUSH.

### Original Communications, Select Observations, etc.

### THERAPEIA.

ART. I.—*Maximum Doses of Medicine.* By Dr. KINGLAKE.

IT would be an acquisition, of the highest importance in medical practice, to be enabled to know, with sufficient precision, how far the doses of medicinal substances might be safely and beneficially extended. Medicines of considerable pretensions, and to which are attached confident expectations, are often given either with ambiguous or inefficient results. When a medical indication for the relief of a pressing malady is clearly afforded, it is deplorable not to be able to meet the occasion with an adequate remedy. In these instances, numerous agents, possessing reputed power, are at hand; but, on trial, they are, too generally, found to be incapable of effecting the desired purpose. Pharmacologists have elaborately arranged the alledged medical experience, as well as the speculations of physiology and pathology, on the virtues and doses of medicines; but still a lamentable deficiency is known to exist on this momentous subject.

Much difficulty must necessarily attend every attempt to generalize the medicinal effects of agents in the animal economy. The differences occurring in the constitutional organization, and in its temperamental influence, are too extensive to be defined. Strict resemblances in any of the arrangements of matter, are rather imaginary than real; some circumstances, either of an integral or adventitious nature, present, to induce a greater or less degree of dissimilarity. *Fac-similes* may be attempted by art, but they are no where found in nature. If, then, it be proposed, that a given medicine should have a certain effect in medical practice, reference should be had to the diversity of vital organization and power on which it is to be exerted. General effects may be reasonably presumed; but particular influence cannot, with any certainty, be anticipated. In the inevitable uncertainty of medicinal agency, an ample scope should be afforded for determining the absolute and relative extent of doses. The nomenclature of diseases has unquestionably been a lamentable stumbling-block in correctly distinguishing the real nature of occurring maladies, and in ascertaining the most appropriate treatment. Too much contrivance and factitiousness has been employed in nominally designating morbid affections, to afford any thing like an accurate and truly practical delineation of natural appearances. The same fallacious attempt at natural correctness is shown in the pharmacopœial definitions that have been given of the qualities, doses, and uses, of medicinal substances. The inquiry should be thrown open, and stand over, for wider and more adequate experience to decide on the general and particular powers of medicinal agents, and to what extent they should be severally administered, to fulfil the various remedial intentions which they are, respectively, designed to effect.

The extreme limit to which a medicine may be carried, would be its *maximum* dose; and this can only be correctly ascertained by gradual augmentation. An extreme dose of medicine, in the first instance, is not well borne; the excitability of the stomach, and that of the system at large, will not endure a strong impression, abruptly made, without inducing a state of re-action that would impede and defeat the effect meant to be produced. By gradually accustoming the excitable power that is to be acted on, whether of the stomach or of any other vital organ, to the particular agency intended to be exerted, it might be made tolerable to an extent that would prove effective in the way designed; whilst less caution, and more precipitancy, in administering the agent, might occasion early disagreement, which would probably render subsequent trials, with a view to eventual benefit, wholly abortive. Distempered excitability, whether from ordinary causes of morbid influence, or from medicinal agency, is not speedily appeased, but remains until the disordered effects shall have been gradually overcome by the returning actions of health. Pending this interval, the medicine which had induced the disturbance could not be used, without inflicting additional injury. It is, therefore, of the utmost importance, in investigating the power of a medicinal substance, not to render it intolerable to the stomach, by prematurely giving it in a dose which would offend, but which, by progressive increase, might, at a later period, have proved a grateful and an effectual remedy.

Arsenic, hydrocyanic or prussic acid, iodine, the nitrate and muriates of quicksilver, the muriates of lime and barytes, tartarized antimony, the animal and vegetable alkalies, the mineral acids, belladonna, digitalis, stramonium, colchicum, elaterium, nux vomica, the hellebores, and many other of the more active medicines, might, by slow introduction into the system, be made remedial in numerous obstinate and threatening ailments; whilst, in a more rapid attempt to render them availing, much and serious mischief might be produced, by suddenly and inordinately affecting the various excitable powers of life. It would, indeed, be an enviable desideratum to know the precise maximum, or largest, dose of medicine, in all conceivable cases; but the very idea of such accurate knowledge pre-supposes an identity of circumstances, never present in either organic or inorganic matter. The excitability of the system is not uniformly the same; it may vary with the day, and even often during that short interval of time: nor are medicinal substances, whether natural or factitious, so rigorously alike as to admit of being regarded as always possessing the same active powers. Absolute doses of medicine, therefore, are less the concern of a cautious and investigating practitioner, than those which are relative. It is required to know, in the particular instance that might occur, what extent of medicinal power might be necessary to effect a given intention of cure. The research is an experiment, and should be guided by scrupulous attention to the effects which might be produced. In some instances, a small dose of medicine may have a full effect: on these occasions, that must be considered as adequate to all that is wanting, and that one of greater strength would disturb and injure, instead of affording the desired relief. At other times, the full extent to which a medicine is accustomed to be given is found to be insufficient; circumstances of an active, though hidden, nature having prevented its usual influence: it, therefore, would be advisable cautiously to augment the power of a medicine, by increasing its dose, until the desired effect be obtained. When this shall have been accomplished, the highest dose, or power, of the medicine, necessary in that particular case, will have been ascertained. This, however, may not be applicable in another apparently-similar case: how far it may be so or not, will be best learnt by pursuing the cautious course of beginning with a small dose, and gradually proceeding to that which may become effective.

The general test of a medicine having been carried to its relative maximum dose, is its offending the stomach, in the first instance, by either pain, nausea, or vomiting; or, ulteriorly, the bowels, the head, or the heart and arteries. If the stomach, rallying from the first impression of nausea, should retain an offending medicine, and transmit its influence, either sympathetically or materially, to the system at large, inducing considerable change in the natural excitement, it would be right to desist from employing it further, lest increasing injury should arise from it; enough having been discovered to admit of concluding that the maximum dose had been exceeded, and that, to persist in it, would be a noxious abuse of its virtues in a more limited quantity. Medicines intended to operate on the first passages are direct in their effect. If the stomach be to be moved by nausea, or more strongly excited by vomiting, either result should speedily ensue the medicine used for the purpose. If, however, neither nausea nor vomiting should follow, but severe pain, the agent will have produced an impression that could not be safely increased: augmented doses, under such circumstances, would be more likely to induce inflammation, than the specific action intended. Similar observations are applicable to the bowels, in reference to the agency of cathartic medicine. The dose, as in the instance of nauseating or emetic operation, might be pushed the length of occasioning some degree of pain; but then it should be to a measured extent, and not be carried far enough to endanger the production of inflammatory excitement.

Medicines intended specifically to affect vital organs beyond the first passages, must, in their respective doses, be subordinate to the excitable conditions of the parts to which they are destined. If it be proposed to produce a diuretic effect, the medicine given for that purpose may be gradually increased in its dose until it be accomplished, provided that the stomach and bowels, through which it must pass, be not painfully and hurtfully disturbed in its transit through them to the system. Various agents, conjoined and given in the same intention, may be sometimes carried further, and rendered more efficient for specific medicinal purposes, than when the desired influence is either exclusively attempted by one article, or restricted to a few multifarious compositions, if simple and precise in its intention, if coincidence of quality and co-operation of power be regarded, is often more effectual and salutary than when less compounded. This fact is strikingly exemplified in the action of purgative medicine. A more decided and powerful effect is produced from a well-assorted variety of cathartic substances, in the same aggregate dose, than from one or two articles of that description. It would appear as if each substance exerted its specific power, and that the union of the whole became more certainly operative than if a less-compounded medicine had been more distinctly active. In the former case, the result would be specifically purgative; it would not exceed the precise limits that would call into increased action the peristaltic power of the intestines: whilst, in the latter, the effect may be more than of a simple stimulant, an excess of which might rather induce inflammatory than cathartic excitement.

The influence of all medicine intended to act in a specific manner,—that is to say, to affect one part of the frame more than another,—may perhaps be improved, both in power and certainty of effect, by combining kindred articles together. The extent of dose would be the same as if but one substance were to be given: even less of the compound would be often found to act more powerfully, because the united whole would exert a similar mode of agency. It is well known that the various excitable powers of different parts of the system are specifically acted on by certain substances. It belongs to experience to ascertain the relative powers subsisting between certain parts of the frame, and the active properties of given agents. The general influence of active substances on the system is that of simple excitement, which is as applicable to one part as to another. If the stomach be simply excited, the effect may be sympathetically extended throughout the whole system, without any organ or particular texture especially arresting its influence; but, if it be of a specific kind,—if it be peculiarly adapted to affect either the kidneys, lungs, liver, brain, or any other organ,—such specific excitement may arise. The property that enables the agent to exert a certain effect in a particular organ has no sensible influence; or, if any, that of a simple stimulus, in those situations where there is no organic adaptation or physical aptitude for giving effect to its power. The simple, as well as specific, action of medicine greatly depends on the dose in which it may be given. It is, therefore, of the utmost practical importance to ascertain how far medicinal substances may be carried in the various intentions in which they are used, to accomplish what may be expected from them. As has been already observed, a great latitude must be allowed to occurring difficulties, both in the excitability to be acted on, and in the power of the agent that may be employed. Precise similarity of effect cannot be confidently expected; but an approximation to it may be reasonably presumed. By slow augmentation, and varying the trial according to the general and particular excitability of the system, a tolerably accurate conclusion may be attained as to how far a medicine, whatever may be the description of its power, may be urged, to enable it to produce its fullest and most salutary effect.

It is obvious that no absolute standard can be laid down as to medicinal doses, in the various and dissimilar states of excitable power, in different, and even the same, constitutions at different times. It would be wrong, indeed, to attempt to control the powers of nature by restrictions, which occurring diversities must ever disown and render unavailing. Medical inquiries, like all other researches, should remain open; new lights must continue to be shed on the obscure and equivocal powers of medicinal efficiency, until, by attentive observation, facts will at length be sufficiently accumulated to serve as a secure guide to farther speculations on the subject.

It has been too much the prevailing practice to direct accredited medicinal articles, in the several intentions in which they are used, to a given extent; and, if the desired effect shall not have been produced, to impute the failure rather to the obstinacy of the disease than to the insufficiency of the medicine. This is taking for granted what is not proved. A more investigating and instructive course would be to extend the dose of the medicine as far as it might be borne, either by the stomach, heart, or brain. If the stomach should reject it, or be painfully affected by its stimulant impression,—if the bowels should be disordered, the heart quickened in its action, or delirious disturbance produced in the brain,—the medicine should not be carried farther, in pursuit of a speculative benefit, in defiance of so much hurtful agency. To attempt to relieve by such violent means, would be to render the remedy as bad, or even worse, than the disease intended to be removed. Until, however, some proof, derived from injurious visceral excitement, shall have been afforded, the maximum dose cannot be said to have been satisfactorily ascertained. Powers of the most remedial description may be possessed by medicines at different degrees of strength, between the limits which insufficient inquiry has imposed on medicinal substances, and the extent to which they should be carried to render them adequately efficient. It is this *terra incognita*,—this unexplored scale of medicinal efficiency, that requires to be freely investigated and made known. Diseases abound,—reputed remedies are also apparently sufficient,—scarcely any malady is without its notional antidote; but fallacy and disappointment are still the common results of curative endeavours. Whilst deficiencies exist, they should be supplied, and every opportunity should be embraced of furnishing what may be wanting in medicinal power, until the reproach of insufficiency be removed, and comparative perfection be imparted to the resources of the healing art.

*Taunton; November 20th,* 1821.

