# Errata in Dr. Kinglake's Paper, Inserted in the Last Number

**Published:** 1822-03

**Authors:** 


*Errata in* Dr. KINGLAKE's *Paper, inserted in the last Number.*

Page 92, lines 30-31, for restricted to *a few*, read restricted *to less.*
— — line 31, for precise in *its* intention, read in intention.
— — — 33, for *is* often, read *they are* often.
— — — 45, for *than*, read *that*.
— 93, — 31, for *difficulties* read *differences.*

☞ Dr. KINGLAKE will perceive how readily we have inserted the above *Errata*, with which he has favoured us; but, in justice to ourselves and to our printer, it should be stated, that four out of the five are *verbal amendments*, made by the author since his manuscript was conveyed to us; and not *errors* of the press.—A. B. G.

